# Supplementary material for: Ultra-sensitive monitoring of leukemia patients using superRCA mutation detection assays
Source: Nat Commun. 2022 Jul 12;13:4033. doi: 10.1038/s41467-022-31397-y (PMC9276831; doi:10.1038/s41467-022-31397-y)
Supplement: Supplementary file 1 — supplementary information [file 41467_2022_31397_MOESM1_ESM.pdf]

Title: Ultra-sensitive monitoring of leukemia patients using superRCA mutation detection assays

Author List: Lei Chen[1,4*], Anna Eriksson[2], Simone Weström[1], Tatjana Pandzic[1], Sören Lehmann[2], Lucia Cavelier[1,3], Ulf Landegren[1*,3]

Affiliations:

[1]Department of Immunology, Genetics and Pathology, Science for Life Laboratory, Uppsala University, SE-752 37 Uppsala, Sweden

[2]Department of Medical Sciences, Uppsala University, SE-751 05 Uppsala, Sweden

[3]These authors jointly supervised this work: Luica Cavelier and Ulf Landegren.

[4]Present address: Rarity Bioscience AB, SE-752 37 Uppsala, Sweden

*Correspondence to:

Ulf Landegren, E-mail: ulf.landegren@igp.uu.se

Lei Chen, E-mail: lei.chen@raritybioscience.com

Homepage: http://www.igp.uu.se/research/molecular_tools/ulf_landegren/

Supplementary material

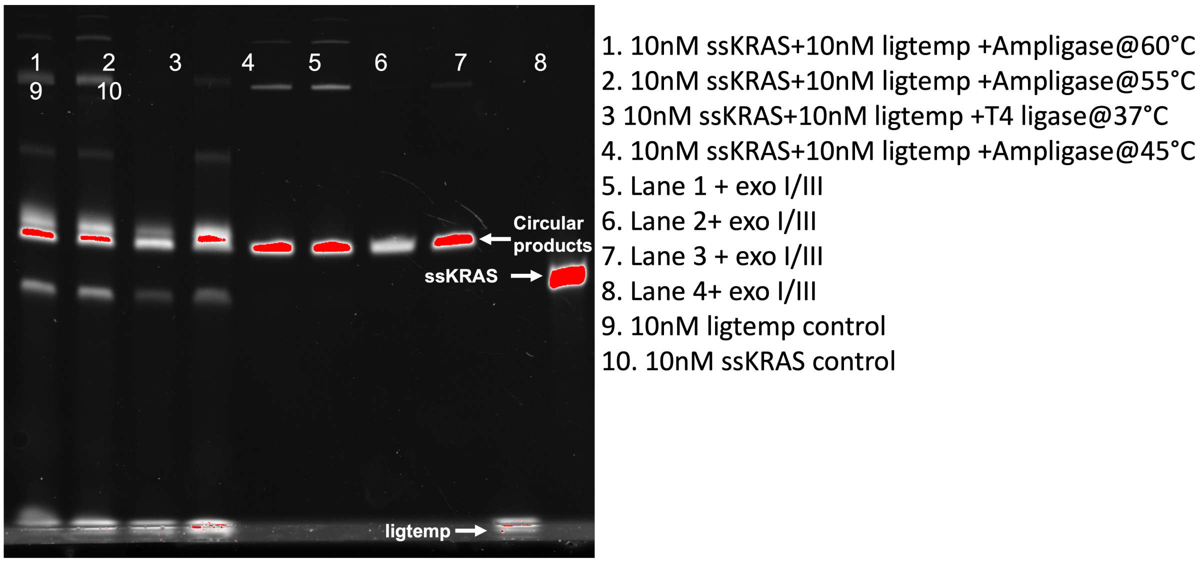

1. 10nM ssKRAS+10nM ligtemp +Ampligase@60°C
2. 10nM ssKRAS+10nM ligtemp +Ampligase@55°C
3 10nM ssKRAS+10nM ligtemp +T4 ligase@37°C
4. 10nM ssKRAS+10nM ligtemp +Ampligase@45°C
5. Lane 1 + exo I/III
6. Lane 2+ exo I/III
7. Lane 3 + exo I/III
8. Lane 4+ exo I/III
9. 10nM ligtemp control
10. 10nM ssKRAS control

Supplementary Figure 1: Analysis of the ligation of linear DNA strands into circles. In the superRCA protocol one strand of the starting PCR product is converted to a DNA circle by templated ligation. Because of the many PCR copies there is no need for 100% ligation efficiency, however we demonstrate in this figure that linear DNA circles can be efficiently converted to DNA circles. In order to be visible by gel electrophoresis with Sybr Gold we used a concentration of 10 nM linear synthetic oligonucleotide with the same sequence as the PCR strand interrogated for KRAS mutations. This concentration is substantially higher thatn the approximately 0.2 pM concentration of PCR products used in the superRCA protocol 1st ligation step. Despite this higher concentration intermolecular ligation to form dimers is negligible. 10 nM linear single strands and 10 nM ligation template were mixed in 1X Ampligase or T4 DNA ligation buffer. The mixture was incubated at 45/55/60°C for 1 hour with 5 U Ampligase (Lane 1/2/4) or 1 U T4 DNA

ligase in Lane 3. After the ligation step, part of the mixture from Lanes 1-4 were treated with 20 U exoI and 200 U exoIII to degrade all the linear single and double stranded molecules, leaving only the single stranded DNA circles intact. The mixture was visualized on a 10% PAGE gel stained with 1X Sybr Gold, demonstrating efficient conversion to DNA circles.

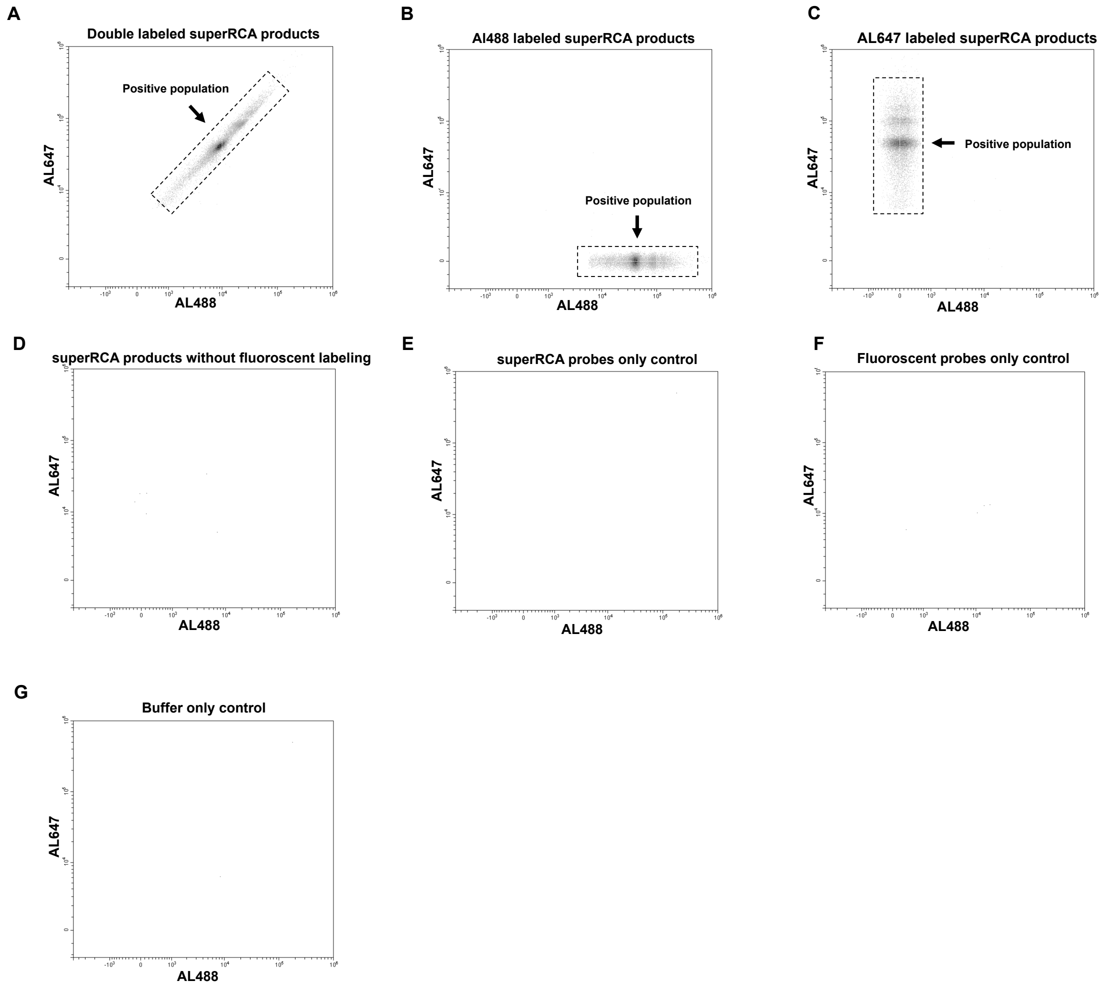

Supplementary Figure 2: Flow cytometric detection of superRCA products and controls. A) Two different padlock probes detecting the same target sequence in primary RCA products were used to generate superRCA products. Secondary RCA products of circularized padlock probes products were

visualized with both AL488- and AL647-conjugated detection oligonucleotides. B) Only padlock probes whose RCA products could be detected by AL488-probes were used to generate the superRCA products. C) Only padlock probes whose RCA products could be detected by AL647-labeled probes were used to generate the superRCA products. D) Both AL488- and AL647-detectable padlock probes were used to generate the superRCA products, but no Al488- or AL647-conjugated oligonucleotides were added. E) Both AL488- and Al647-detectable padlock probes and their detection oligonucleotides were added, but no first-generation RCA products was present to act as ligation templates for the padlock probes. F) Only AL488 and AL647 labeled detection oligonucleotide were present in the sample. G) only reaction buffer presented in this sample.

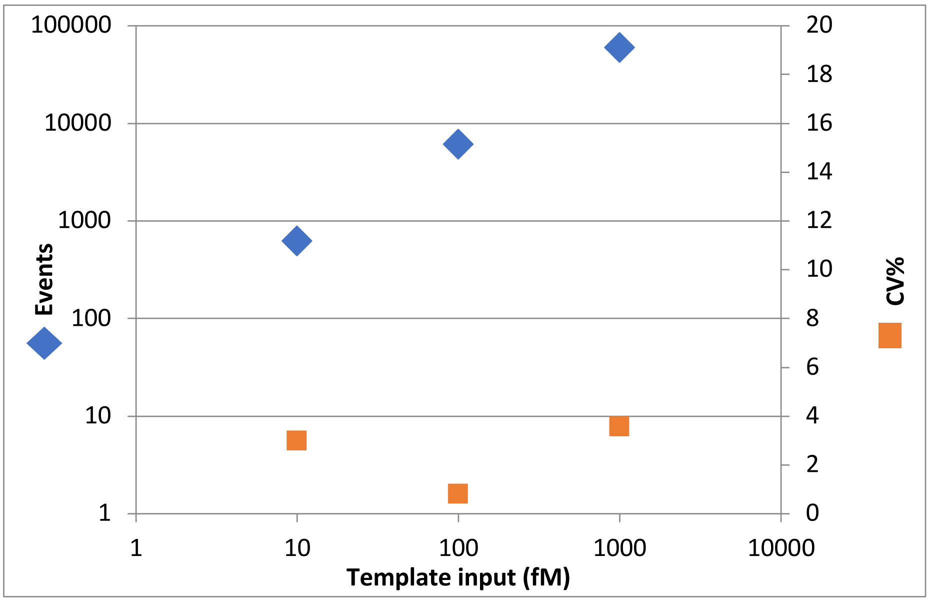

Supplementary Figure 3: Counting of superRCA products via flow cytometry. Coefficients of variation of results of the flow cytometry counts. The blue diamonds represent the average numbers of

counted events for the different template input, and the red squares represent the calculated CV value for the corresponding measurements.

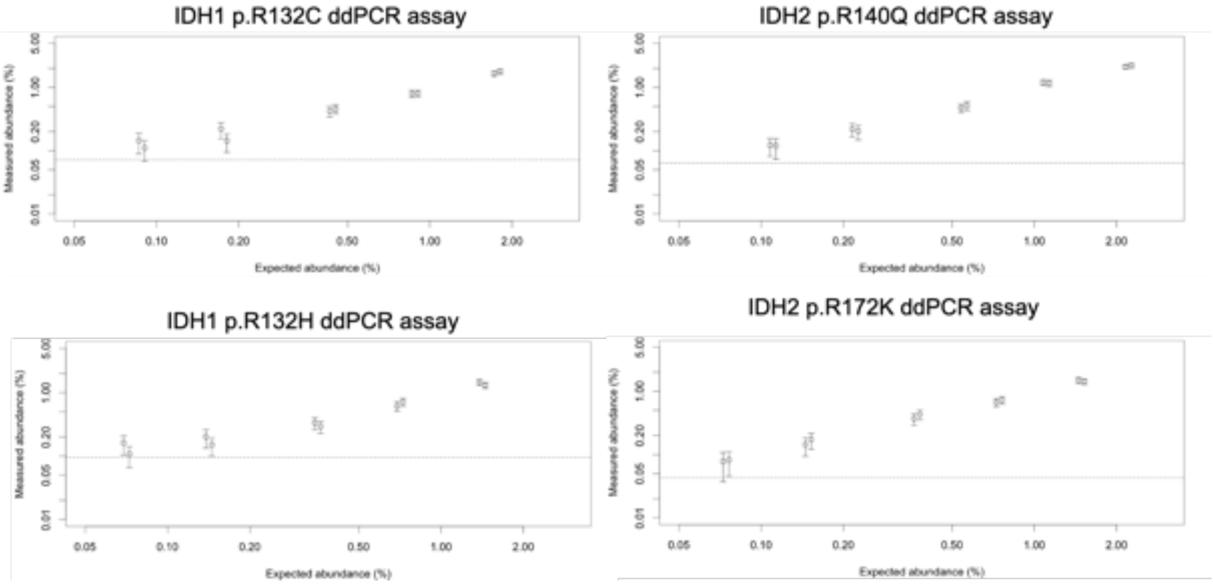

Supplementary Figure 4: Mutation detection performance of commercial Bio-Rad ddPCR assays for four IDH1/2 mutations with 40,000 copies genome input. Data from the Bio-Rad website, A log-in might be needed to see the details of these pre-validated ddPCR assay.

(https://www.bio-rad.com/digital-assays/assay-detail/dHsaMDV2010055,
https://www.bio-rad.com/digital-assays/assay-detail/dHsaMDV2010057,

https://www.bio-rad.com/digital-assays/assay-detail/dHsaMDS759712575,
https://www.bio-rad.com/digital-assays/assay-detail/dHsaCP2000060)

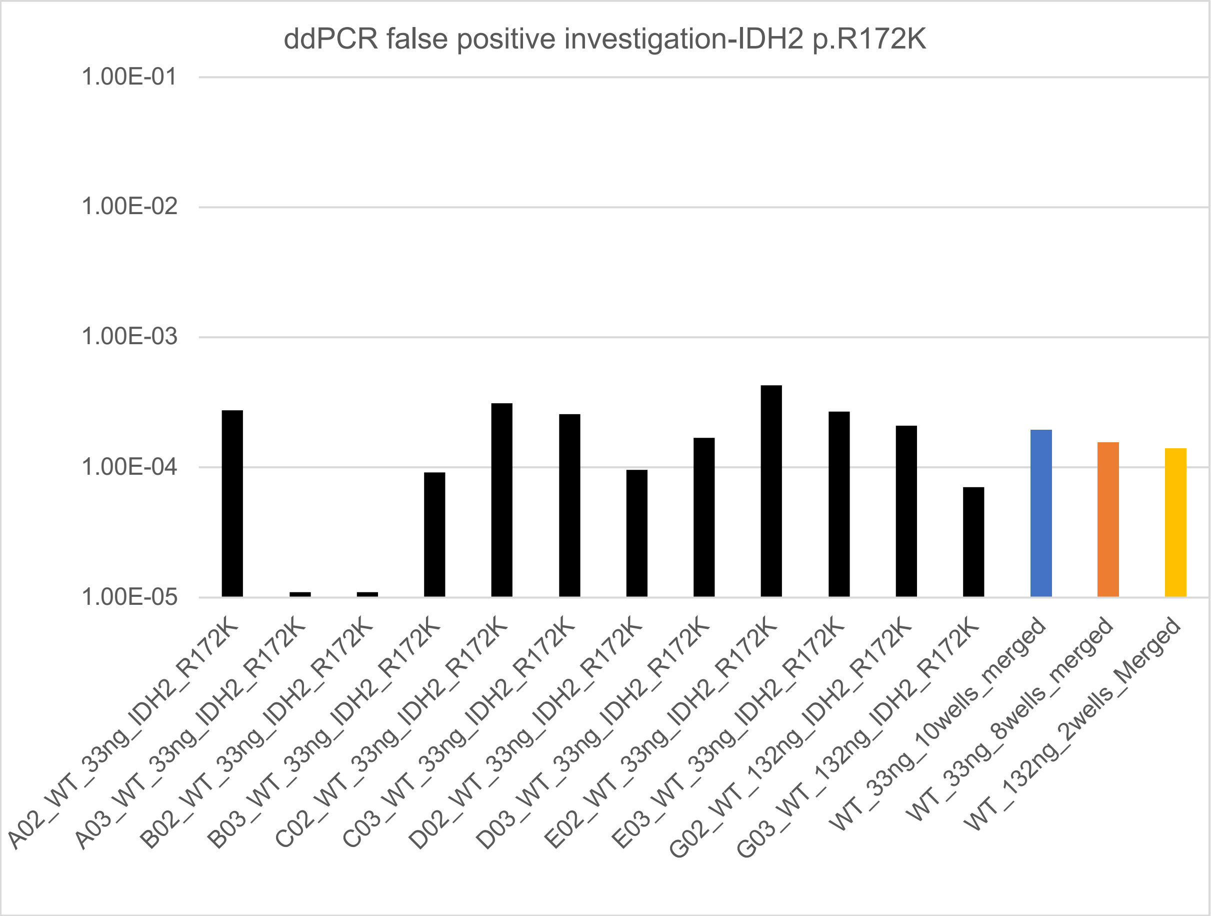

Supplementary Figure 5: Investigation of false positive rate in ddPCR using a wild-type DNA sample. We investigated the ddPCR background levels in wild-type DNA sample using the well-performing ddPCR assay for IDH2 p.R172K in the IDH panel. 33 ng gDNA per well was used in wells A02, A03, B02, B03, C02, C03, D02, D03, E02 and E03. The false positive rate for 33 ng gDNA input condition (A02 to E03) varied from 9.1×10^{-5} to 4.3×10^{-4}, except for A03 and B02 where no false positives were seen. When the gDNA

input in accordance with the manufacturer’s instructions was increased to 132 ng (40,000 haploid genome copies per well) in wells G02 and G03, then the false positive rate remained at 7.0×10^{-5} to 2.1×10^{-4}, rather than decreasing in proportion to the input of gDNA. Furthermore, by pooling 10 wells (blue bar), 8 wells (orange bar) with 33 ng gDNA each (same as in wells A02 to E03) or 2 wells (yellow bar, pooled with G02 and G03) with 132ng gDNA ddPCR, the false positive rates again remained at similar levels. The results demonstrate that the false positive rates in this experiment was not improved by increasing the gDNA input from 33 ng per well.

A)

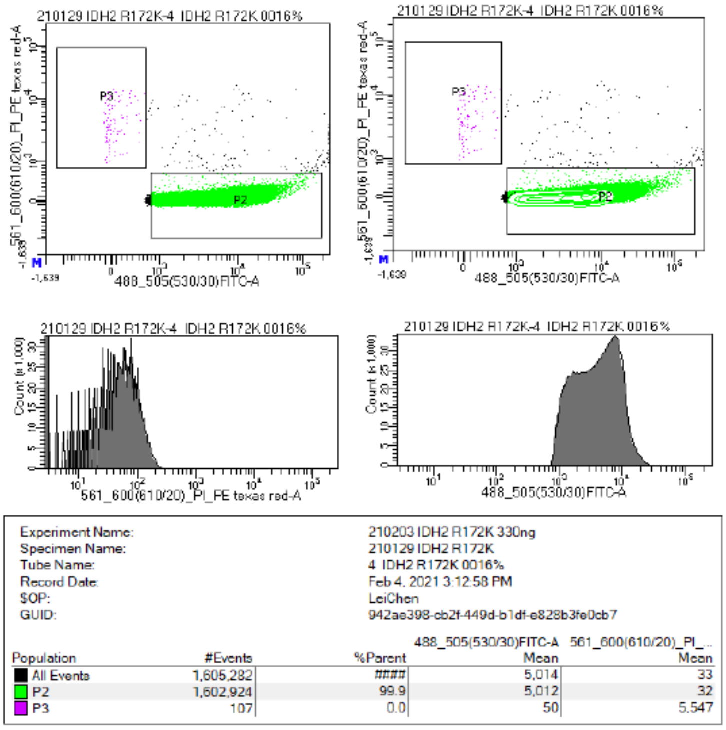

B)

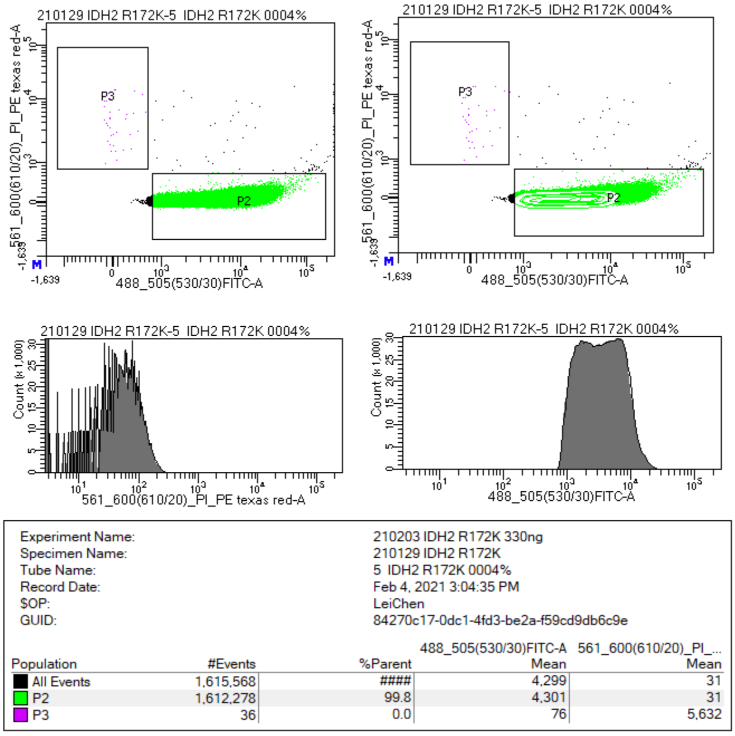

210129 IDH2 R172K-5 IDH2 R172K 0004%
561_600(610/20)_PI_PE texas red-A
488_505(530/30)FITC-A
P3
P2
Count (x 1,000)
Experiment Name: 210203 IDH2 R172K 330ng
Specimen Name: 210129 IDH2 R172K
Tube Name: 5 IDH2 R172K 0004%
Record Date: Feb 4, 2021 3:04:35 PM
SOP: LeiChen
GUID: 84270c17-0dc1-4fd3-be2a-f59cd9db6c9e
Population | #Events | %Parent | 488_505(530/30)FITC-A Mean | 561_600(610/20)_PI_... Mean
All Events | 1,615,568 | #### | 4,299 | 31
P2 | 1,612,278 | 99.8 | 4,301 | 31
P3 | 36 | 0.0 | 76 | 5,632

C)

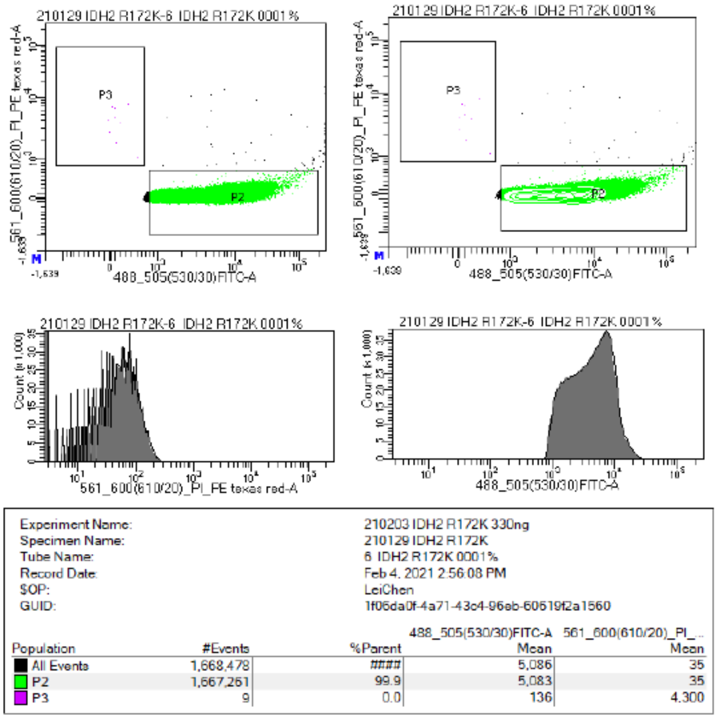

210129 IDH2 R172K-6 IDH2 R172K 0001%
561_600(610/20)_PI_PE texas red-A
488_505(530/30)FITC-A
P3
P2
Count (x 1,000)
Experiment Name: 210203 IDH2 R172K 330ng
Specimen Name: 210129 IDH2 R172K
Tube Name: 6 IDH2 R172K 0001%
Record Date: Feb 4, 2021 2:56:08 PM
SOP: LeiChen
GUID: 1f06da0f-4a71-43c4-96eb-6061912a1560
Population | #Events | %Parent | 488_505(530/30)FITC-A Mean | 561_600(610/20)_PI_... Mean
All Events | 1,668,478 | #### | 5,086 | 35
P2 | 1,667,261 | 99.9 | 5,083 | 35
P3 | 9 | 0.0 | 136 | 4,300

D)

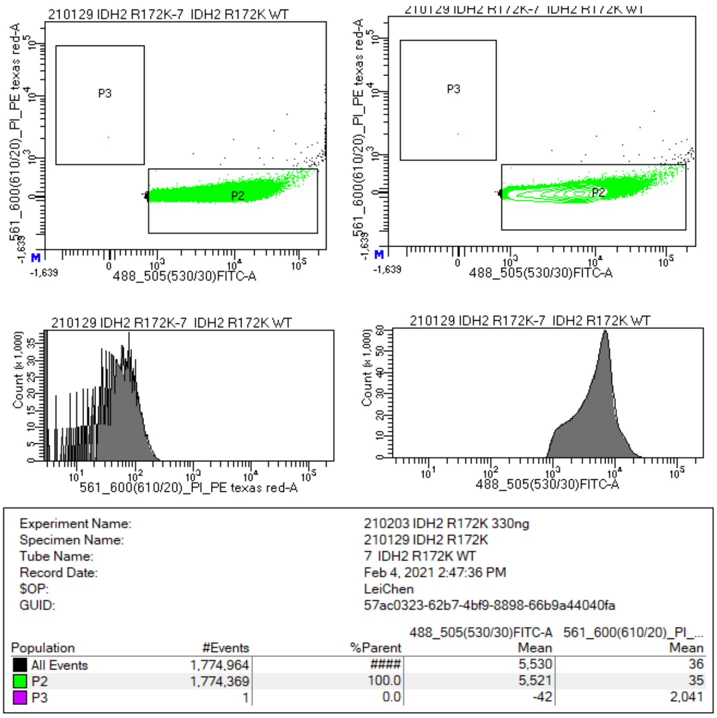

Population	#Events	%Parent	488_505(530/30)FITC-A Mean	561_600(610/20)_PI_... Mean
All Events	1,774,964	####	5,530	36
P2	1,774,369	100.0	5,521	35
P3	1	0.0	-42	2,041

E)

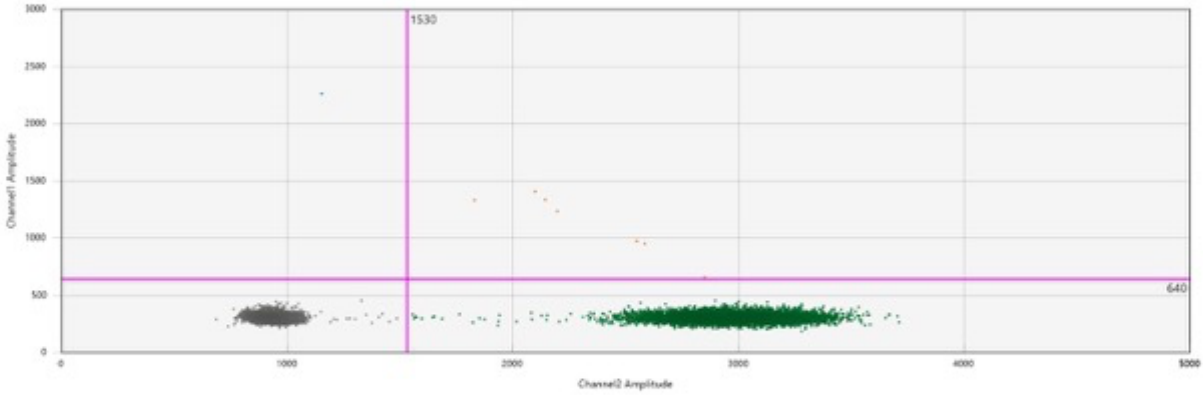

F)

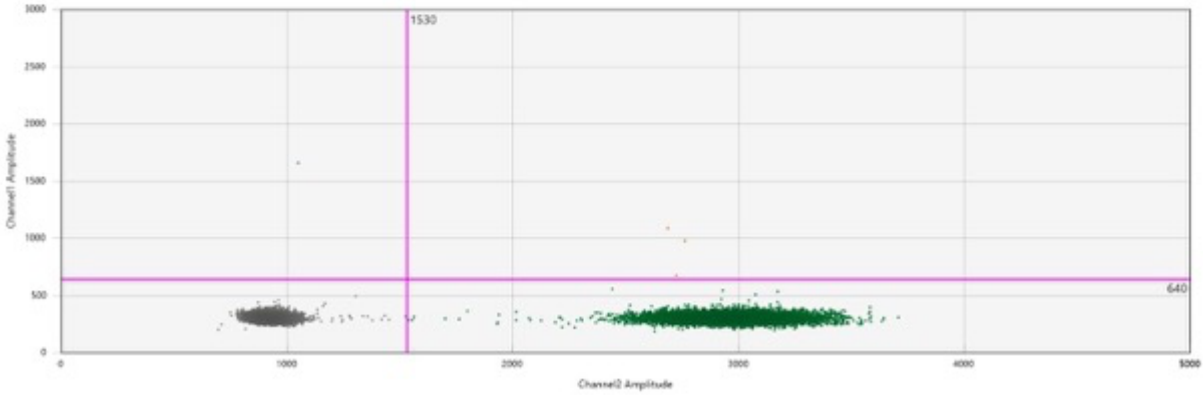

G)

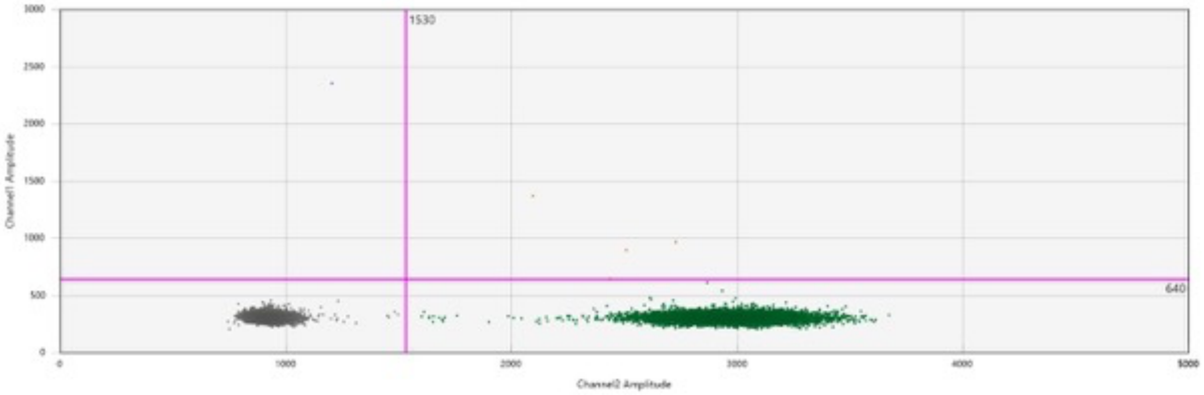

H)

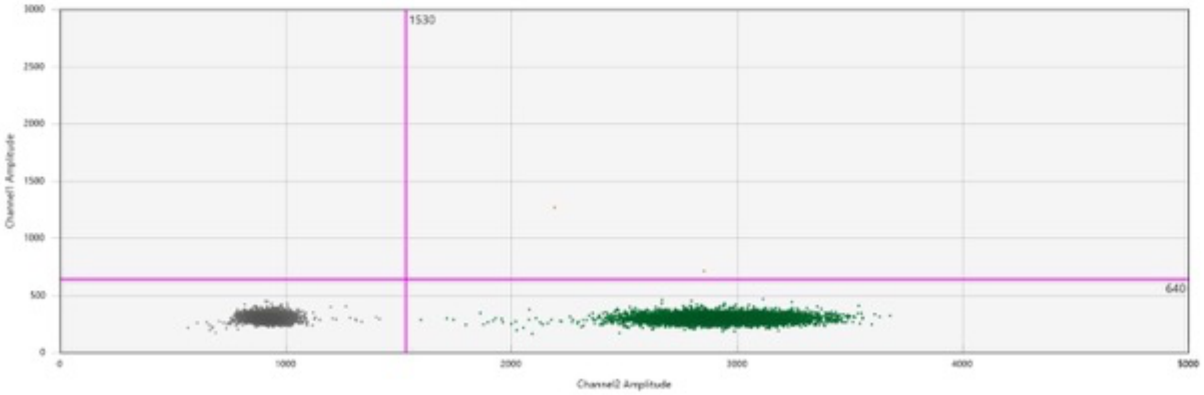

Supplementary Figure 6: Raw data for superRCA and ddPCR analyses of samples with the IDH2 p.R172K mutation spiked at low frequencies and for wildtype samples. A) superRCA flow raw data for the IDH2 p.R172K mutation spiked at 0.016%. B) superRCA flow raw data for the IDH2 p.R172K mutation spiked at 0.004%. C) superRCA flow raw data for the IDH2 p.R172K mutation spiked at 0.001%. D) superRCA flow raw data for a wild type sample. E) ddPCR raw data for the IDH2 R172K mutation spiked at 0.016%. F) ddPCR raw data for the IDH2 R172K mutation spiked at 0.004%. C) ddPCR raw data the IDH2 R172K mutation spiked at 0.001%. D) ddPCR raw data for a wild type sample.

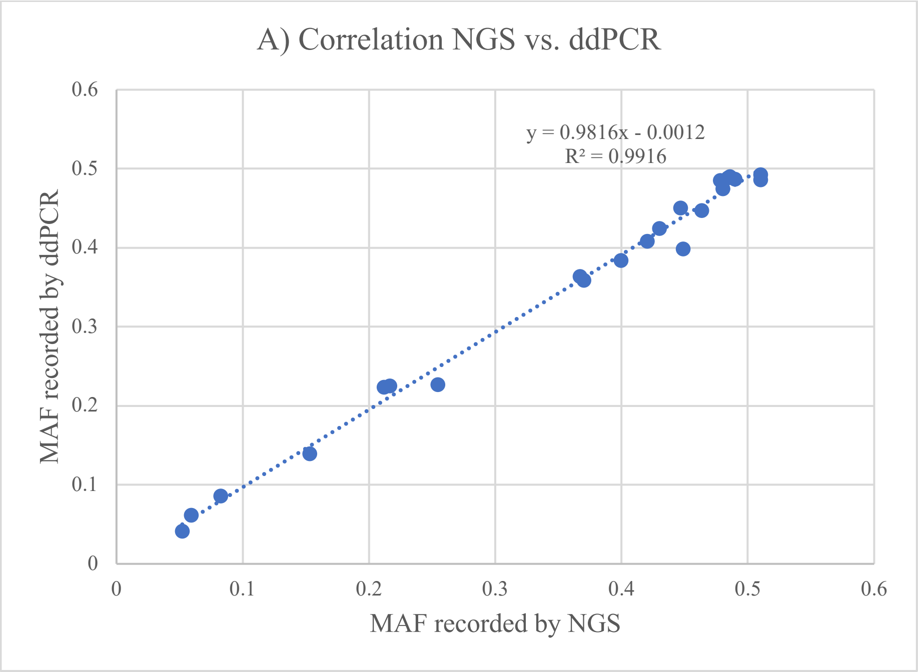
A) Correlation NGS vs. ddPCR
y = 0.9816x - 0.0012
R² = 0.9916
MAF recorded by ddPCR
MAF recorded by NGS
0
0.1
0.2
0.3
0.4
0.5
0.6

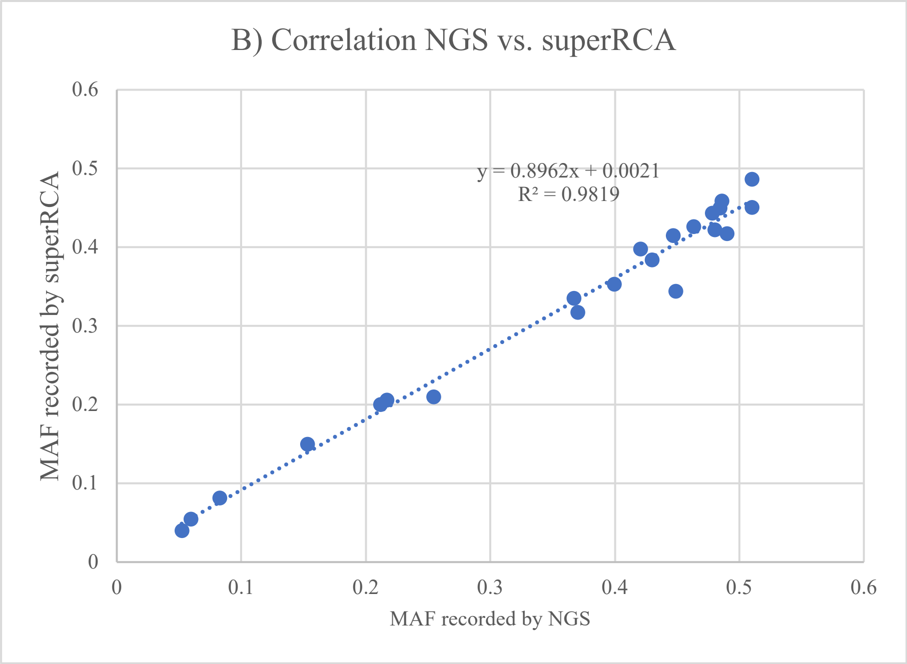
B) Correlation NGS vs. superRCA
y = 0.8962x + 0.0021
R² = 0.9819
MAF recorded by superRCA
MAF recorded by NGS
0
0.1
0.2
0.3
0.4
0.5
0.6

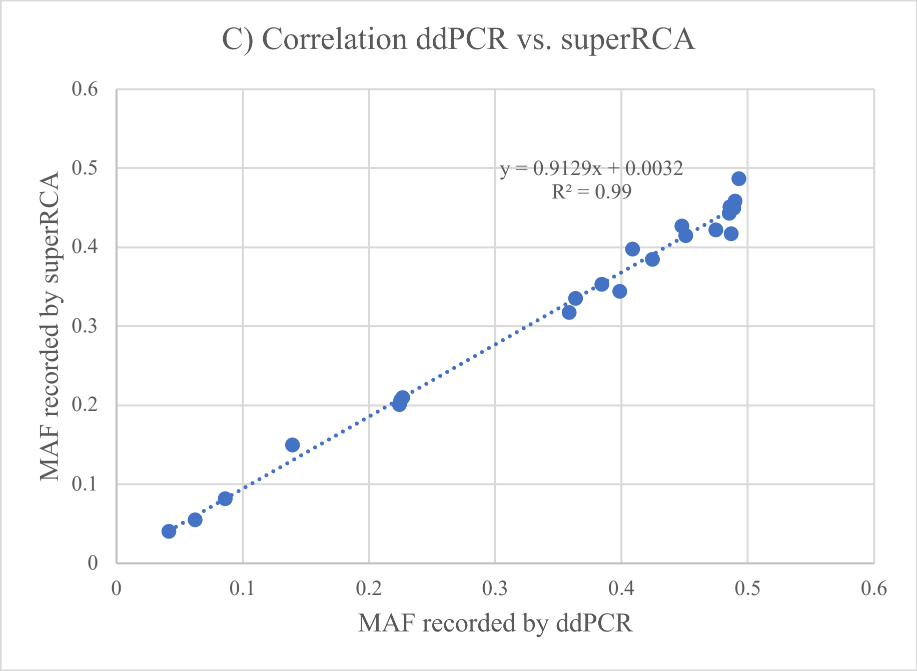
C) Correlation ddPCR vs. superRCA
y = 0.9129x + 0.0032
R² = 0.99
MAF recorded by superRCA
MAF recorded by ddPCR
0
0.1
0.2
0.3
0.4
0.5
0.6

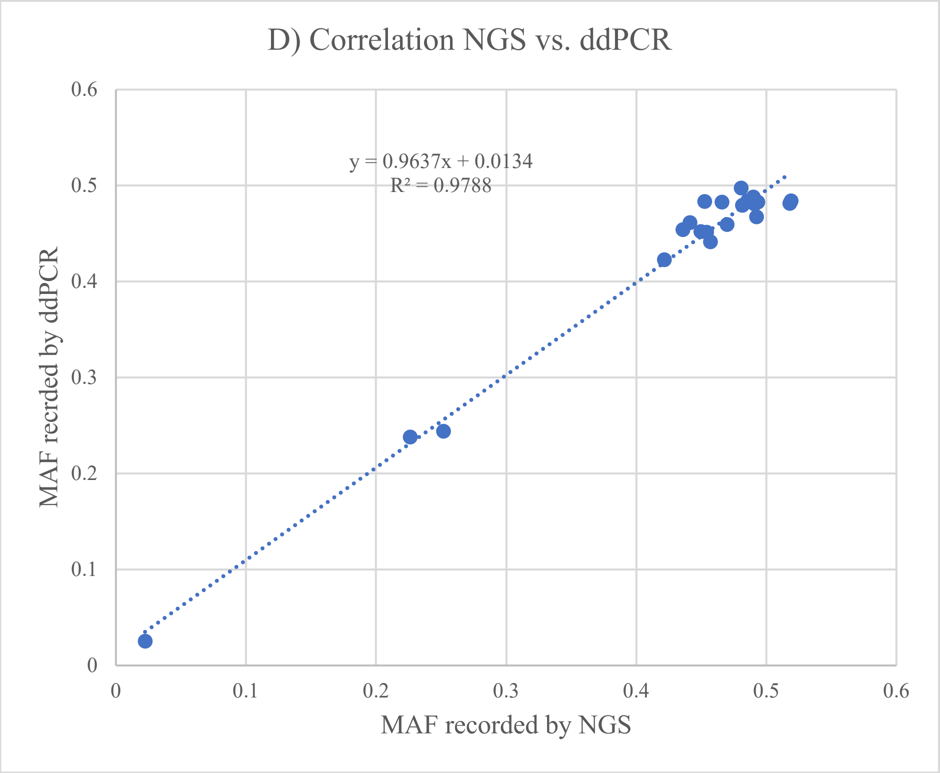
D) Correlation NGS vs. ddPCR
y = 0.9637x + 0.0134
R² = 0.9788
MAF recrded by ddPCR
MAF recorded by NGS
0
0.1
0.2
0.3
0.4
0.5
0.6

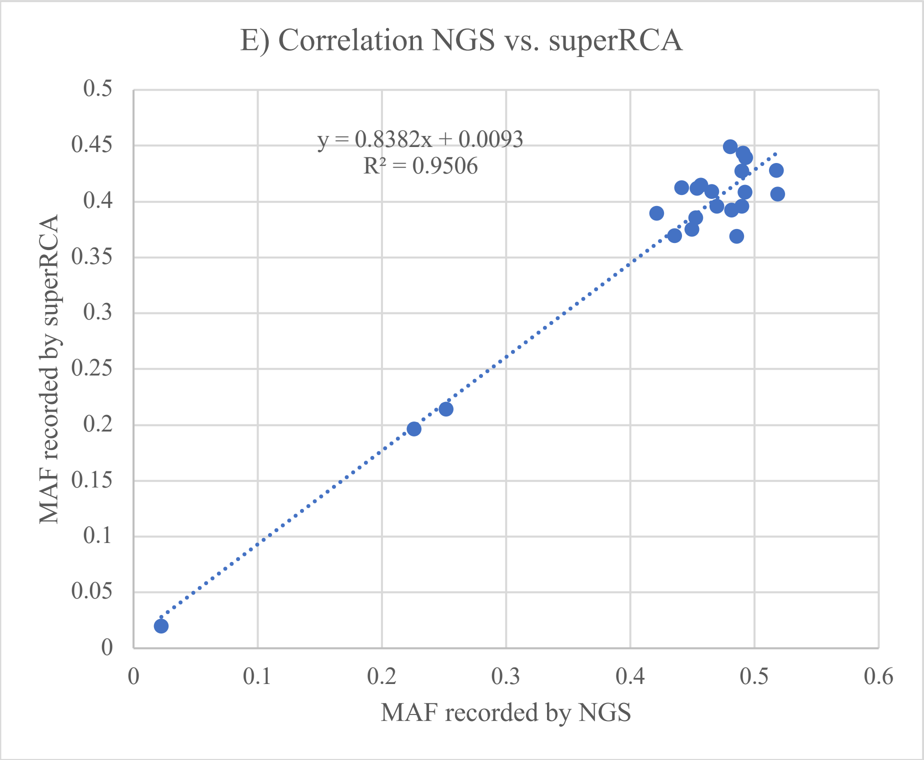

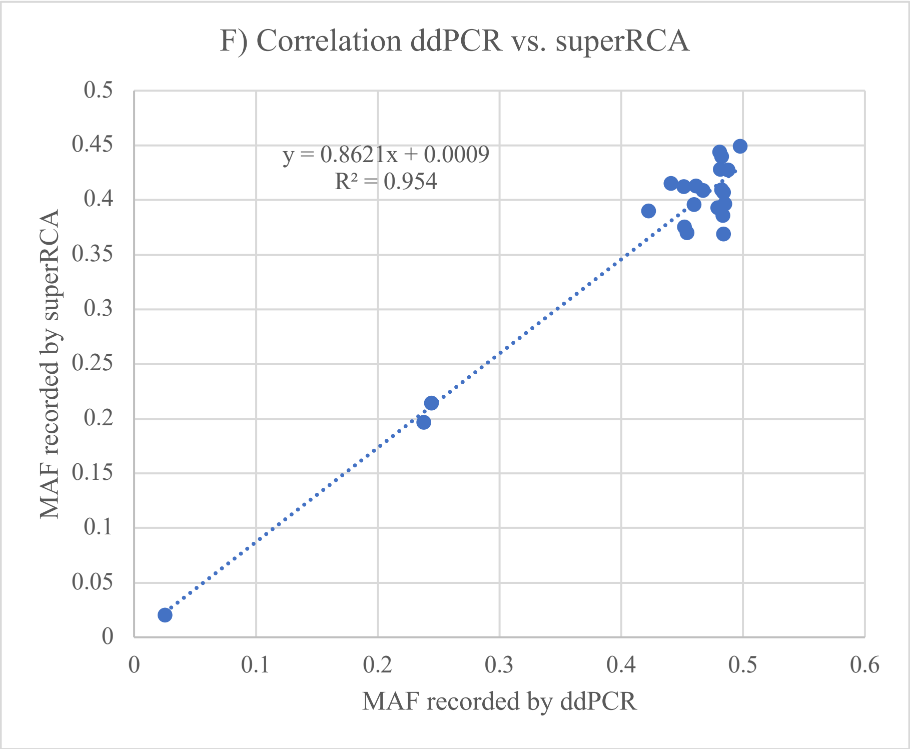

Supplementary Figure 7. Analysis of correlations between results using the three methods to detect MAF – NGS, ddPCR and superRCA – for samples with MAF >0.01. Data for panels A), B) and C) were derived from the results displayed in Figure 5, while those for panels D), E, and F) were from

Figure S6. The superRCA data were reproduced using the same pre-amplified PCR products previously investigated from the initial diagnostic patient samples but diluted 10-fold before flow acquisition (Fig. 5 and Fig. S6)). Diluted superRCA samples were used to resolve the population located in the top right corner of the flow chart, see Figure S11 for the effect of diluting superRCA assay when detecting high MAF samples.

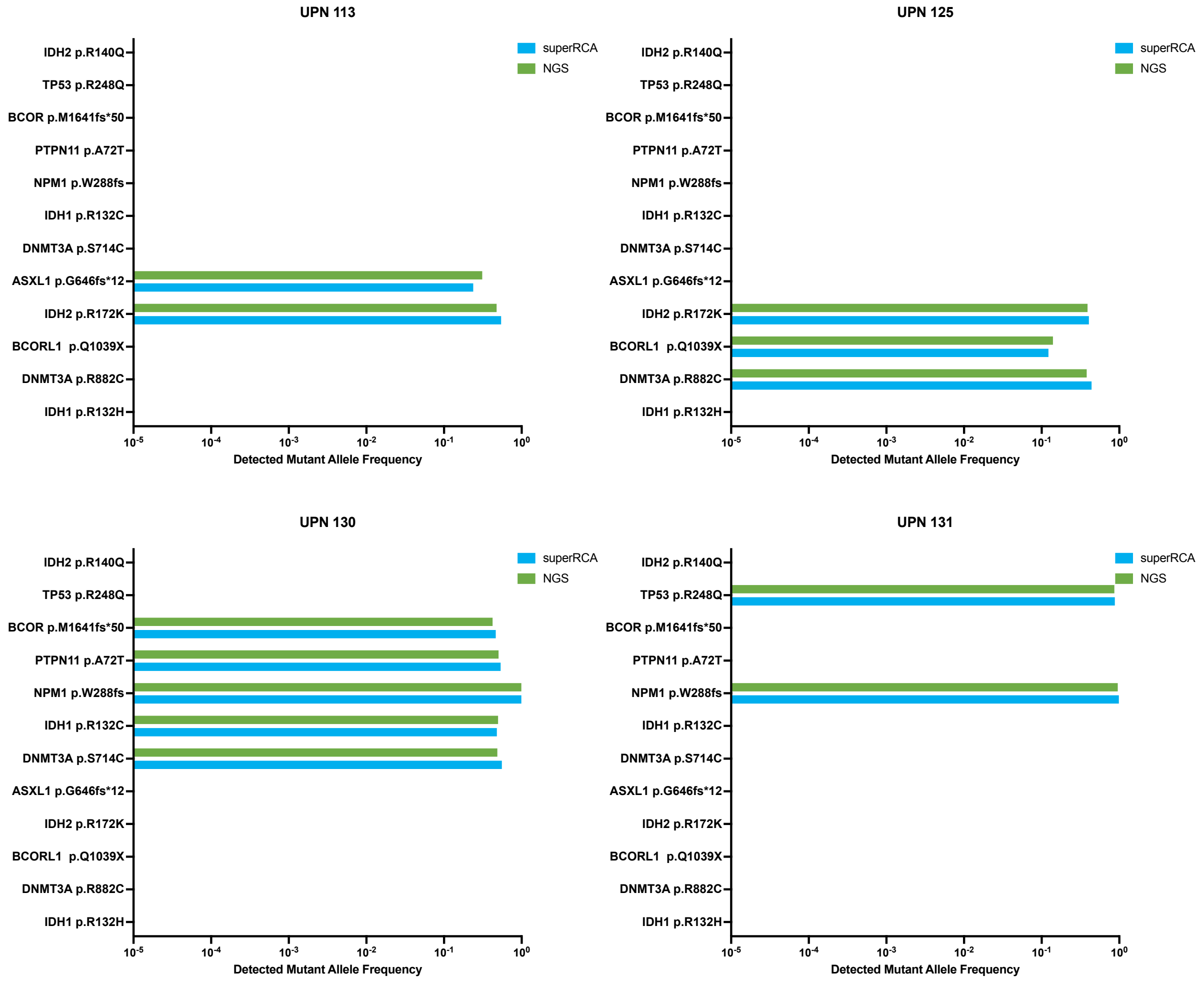

Supplementary Figure 8. Simultaneous detection of multiple mutations in single portion DNA sample. 4 diagnostic AML patient samples were analyzed by the 12-plex superRCA AML

mutation panel with dedicated probes for each mutation. The superRCA analysis results were plotted side-by-side with the NGS analysis data.

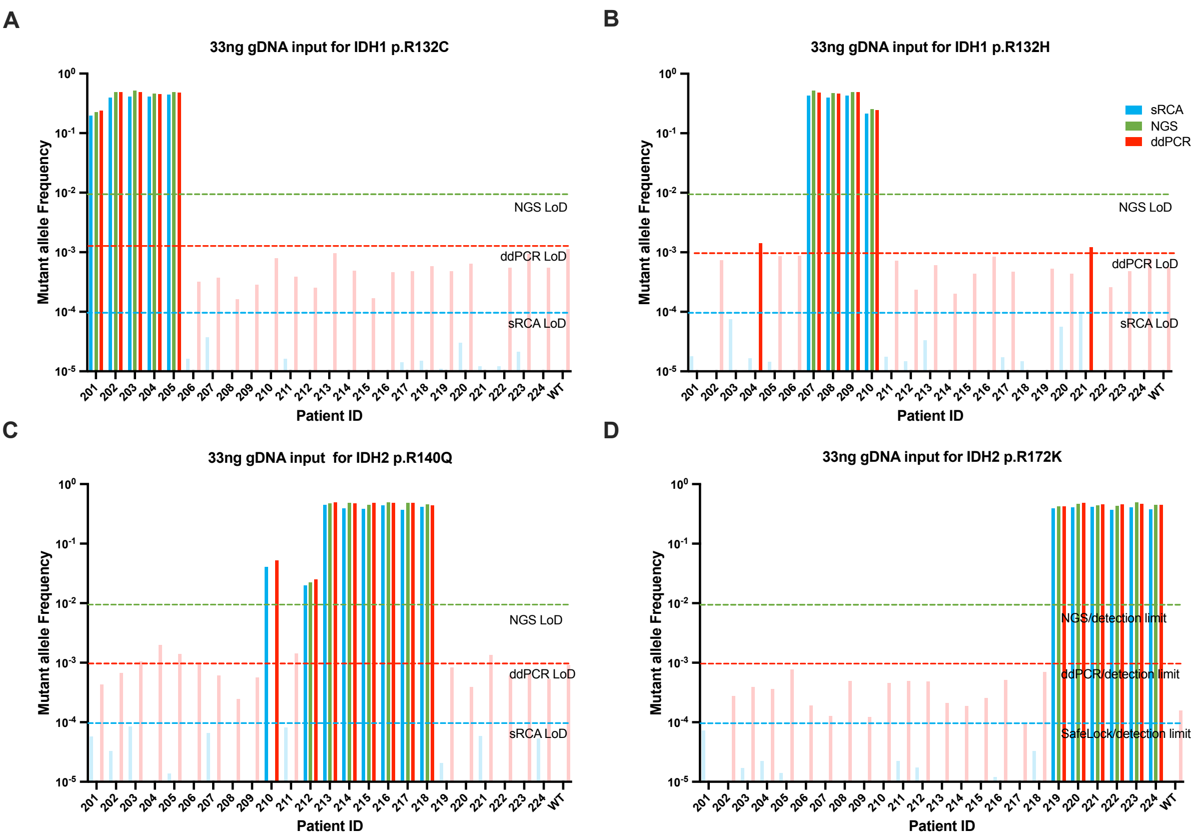

Supplementary Figure 9. Validation of the superRCA, NGS and ddPCR assays in BM aspirates from a second cohort of AML patients. Samples from 24 patients, all with relatively high proportions of malignant cells in their BM, were selected for confirmation of the benchmark study. Here 33 ng genomic DNA was used per patient for the multiplexed superRCA assays. After the pre-amplification step in the superRCA protocol the products were combined for parallel analysis of the four IDH mutations. For the ddPCR assay, four IDH mutation assays were performed per patient, each mutation assay using 33 ng

gDNA divided into two replicates. Samples for all three analyses were collected at diagnosis of the patients.

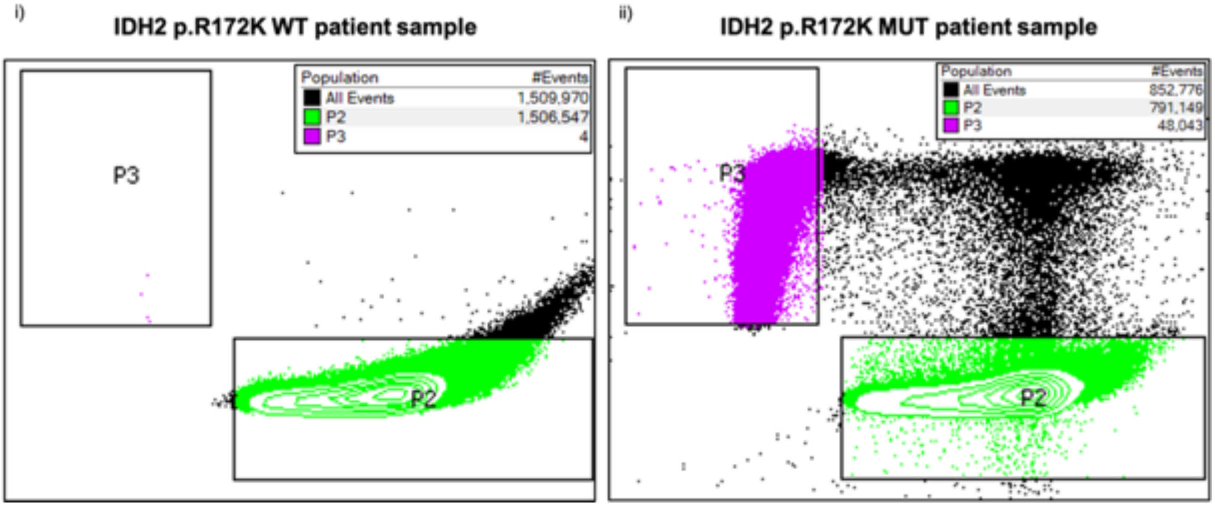

Supplementary Figure 10. Flow cytometric analysis of superRCA products, identifying the IDH2 p.R172K mutant and wildtype IDH2 sequences in genomic DNA samples from two AML patients. Results for a patient whose malignant cells did not carry this particular mutation (i), and for a patient whose BM carried the IDH2 p.R172K mutation (ii). The X axis represents the AL-488 channel used to detect wild-type superRCA products, while the Y axis reflects the AL-647 channel that was used to detect IDH2 p.R172K mutant superRCA products. A small number of mutation-specific events are recorded below the detection threshold and may represent mutations introduced during the PCR step. The polymerase-based target amplification by PCR carries a risk of introducing mutations due to

polymerase errors. Considering the mutation rate of the SuperFi DNA polymerase used for PCR is 300X better than Taq DNA poly error rate[42] ($1.3X10^{-4}$) reported at the vendor's website($4.3X10^{-7}$), 15 cycles of pre-amplification as used herein would entail a $6.45\ x10^{-6}$ risk of introducing a mutation at any specific site. This number is lower than the lowest mutation frequency that could be observed when using a DNA input of 330 ng, corresponding to about 100,000 copies of the haploid human genome. At the high acquisition speed of approximately 10,000 events per second that was used two superRCA products may pass together through the detector at a relatively low frequency. The objects outside the gates and located in the top-right corner in figure S10ii arise when a pair of superRCA products representing both mutant and wildtype sequences have been recorded together. We use this high acquisition speed in order to achieve efficient mutation detection at low frequency ranges (0.001% -1%). As seen in Figure S7, the results of superRCA are in good agreement with both those of NGS and ddPCR. Nonetheless, the occurrence of duplicate events in flow can be reduced by using a lower acquisition speed, or more dilute solutions of superRCA products if desired.

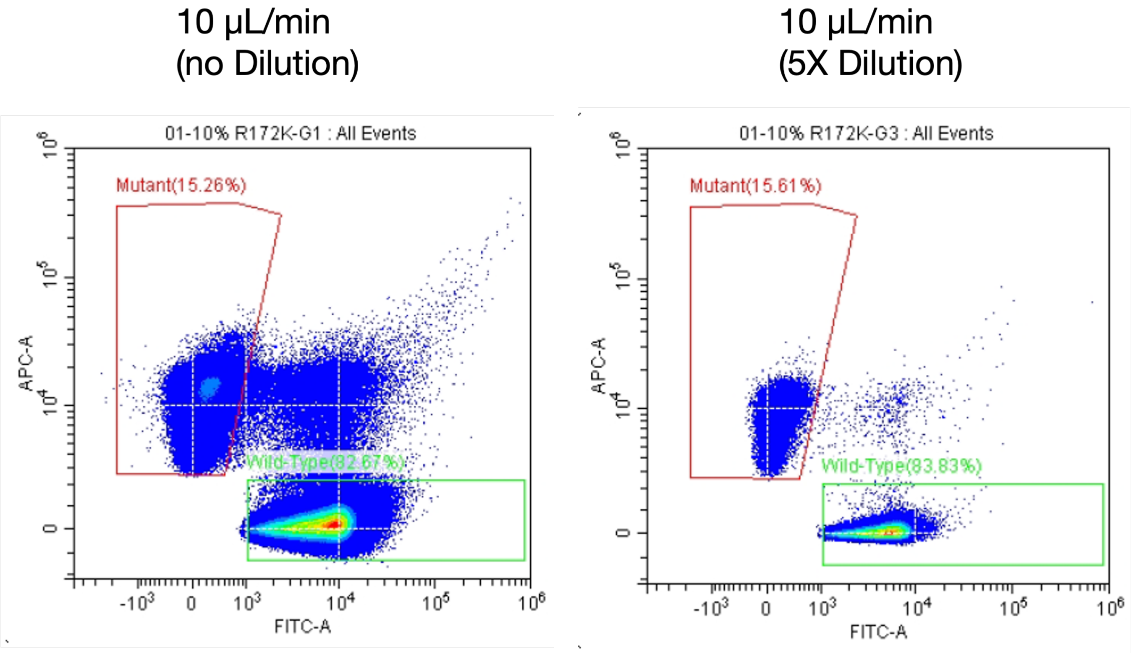

Population	Events	% Total	% Parent	Mean FITC-A	Mean APC-A
All Events	1360409	100.00%	100.00%	5870.2	2268.3
Wild-Type	1124625	82.67%	82.67%	6813.7	223.1
Mutant	207650	15.26%	15.26%	206.9	11941.1

Population	Events	% Total	% Parent	Mean FITC-A	Mean APC-A
All Events	142226	100.00%	100.00%	3988.5	1583.3
Wild-Type	119223	83.83%	83.83%	4677.8	126.8
Mutant	22205	15.61%	15.61%	164.4	9152.4

97.93% in gates

99.44% in gates

Supplementary Figure 11: A patient sample with 16.5% of IDH2 p.R172K mutation was used to investigate the effect of sample concentration during flow readout. Under our standard conditions 98% of the total population of superRCA could be fitted in the gate (left plot). For samples diluted 5X or 10X before analyzing by flow with acquisition at the same speed, around 99.5% of the total population could be fitted in the gate. This demonstrates that the population of superRCA products in the upper right of the scatter plots are due to a low frequency of pairs of mutant and wildtype superRCA products simultaneously passing through the laser when the MAF is high. At MAF below 1%, very few double positives are observed.

Supplementary Table 1: Comparison between some approaches for measuring low-frequency mutations.

Technology	superRCA	BEAMing	ddPCR	Targeted NGS
**Sensitivity**	10e-5	10e-4	~10e-3	~5x10e-3(UMI-)/10e-5(UMI+)
**High-GC performance**	++++	++	+	++
**Mutliplex**	Yes	Yes	Limited	Yes
**Sample → result**	~4h	~36h	~6h	~1 week
**Cost**	+	++	+	+++

Supporting material

Supplementary Note 1: Comparison between an UMI-based NGS approach and the superRCA assay

The superRCA assay requires only 3.5 hr to establish the MAF starting from a purified DNA sample. Using the NGS workflow with TwinStrand the library prep takes 2-3 days (https://view-su2.highspot.com/viewer/620536438e9763f5a86e04bf?iid=6202b0c0d7ae866972f2b220&source=email.620536438e9763f5a86e04c0.0). The sample detection efficiency is the major difference between TwinStrand and superRCA apart from the technical approaches on mutation base calling. Accroding to the Twinstrand technical document, with 500ng DNA input, TwinStrand can detect sinlge mutation at 1 in 3667 (approximately 0.027%), while with superRCA, we can confidently detect mutations at 0.001% with 660ng input in our spike-in data. The superRCA is far more efficient in detecting mutations in limited sample quantity, especially when analyzing ctDNA samples which is known for the low abundance in plasma samples. After the TwinStrand sample prep, a subsequent Illumina MiSeq sequencing reaction takes 65 hours with 600 sequencing cycles considering also the bioinformatic workload (https://support.illumina.com/documents/documentation/system_documentation/miseq/miseq_v3_upgrade_faqs.pdf). Accordingly, there is typically a one week turn-around time using the NGS approach. A Miseq run delivers approximately 20 million single reads at a cost of 1900 USD. For the UMI NGS approach, each UMI needs to be observed in 10 molecules to obtain a consensus

read. To reach a 1 in 100,000 sensitivity it is necessary to allocate 1 million reads per mutation per sample. This would translate to a sequencing cost of 95 USD per mutation. To this should be added the sequencing library preparation cost (the cost of the TwinStrand AML panel library is 722 USD per sample on a 48 sample submission basis (https://www.mdanderson.org/content/dam/mdanderson/documents/core-facilities/sequencing-and-microarray-facility/ATGC%20FY2022%20Price%20Schedule.pdf). The NGS approach has the advantage of detecting any sequence aberrations in the enriched regions. superRCA typically delivers 1-2 million events at a cost similar to that of Bio-Rad ddPCR level at a mutation sensitivity of 1 in 100,000, while the Bio-Rad ddPCR only delivers 20,000 events with a substantially lower mutation detection sensitivity.

Supplementary Note 2: Protein markers for MRD estimation of three AML patients using antibody staining and flow cytometry

UPN 124: CD34+, CD117+, CD33+, CD13 heterogenous HLA-DR+ and a subpopulation CD56+

UPN 125: CD34+, CD117+, CD33 dim, CD13 heterogenous MPO+, morphologic signs of dysplasia

UPN 126: CD34+, CD117+, CD13+, HLA-DR+
