# Supplementary material for: Ultra-sensitive monitoring of leukemia patients using superRCA mutation detection assays
Source: Nat Commun. 2022 Jul 12;13:4033. doi: 10.1038/s41467-022-31397-y (PMC9276831; doi:10.1038/s41467-022-31397-y)
Supplement: Supplementary file 2 — Reporting Summary [file 41467_2022_31397_MOESM2_ESM.pdf]

nature portfolio

Corresponding author(s): Ulf Landegren
Last updated by author(s): May 30, 2022

Reporting Summary

Nature Portfolio wishes to improve the reproducibility of the work that we publish. This form provides structure for consistency and transparency in reporting. For further information on Nature Portfolio policies, see our Editorial Policies and the Editorial Policy Checklist.

Statistics

For all statistical analyses, confirm that the following items are present in the figure legend, table legend, main text, or Methods section.

n/a	Confirmed	
☒	☐	The exact sample size (n) for each experimental group/condition, given as a discrete number and unit of measurement
☐	☒	A statement on whether measurements were taken from distinct samples or whether the same sample was measured repeatedly
☒	☐	The statistical test(s) used AND whether they are one- or two-sided<br>*Only common tests should be described solely by name; describe more complex techniques in the Methods section.*
☒	☐	A description of all covariates tested
☒	☐	A description of any assumptions or corrections, such as tests of normality and adjustment for multiple comparisons
☐	☒	A full description of the statistical parameters including central tendency (e.g. means) or other basic estimates (e.g. regression coefficient) AND variation (e.g. standard deviation) or associated estimates of uncertainty (e.g. confidence intervals)
☒	☐	For null hypothesis testing, the test statistic (e.g. F, t, r) with confidence intervals, effect sizes, degrees of freedom and P value noted<br>*Give P values as exact values whenever suitable.*
☒	☐	For Bayesian analysis, information on the choice of priors and Markov chain Monte Carlo settings
☒	☐	For hierarchical and complex designs, identification of the appropriate level for tests and full reporting of outcomes
☒	☐	Estimates of effect sizes (e.g. Cohen's d, Pearson's r), indicating how they were calculated

Our web collection on statistics for biologists contains articles on many of the points above.

Software and code

Policy information about availability of computer code

Data collection	CytExpert 2.4.0.8
Data analysis	CytExpert 2.4.0.8

For manuscripts utilizing custom algorithms or software that are central to the research but not yet described in published literature, software must be made available to editors and reviewers. We strongly encourage code deposition in a community repository (e.g. GitHub). See the Nature Portfolio guidelines for submitting code & software for further information.

Data

Policy information about availability of data

All manuscripts must include a data availability statement. This statement should provide the following information, where applicable:

- Accession codes, unique identifiers, or web links for publicly available datasets
- A description of any restrictions on data availability
- For clinical datasets or third party data, please ensure that the statement adheres to our policy

The datasets generated during and/or analysed during the current study are available from the corresponding author on reasonable request due to the confidentiality protection of patient related information in the raw data.

Field-specific reporting

Please select the one below that is the best fit for your research. If you are not sure, read the appropriate sections before making your selection.

[x] Life sciences [ ] Behavioural & social sciences [ ] Ecological, evolutionary & environmental sciences

For a reference copy of the document with all sections, see nature.com/documents/nr-reporting-summary-flat.pdf

Life sciences study design

All studies must disclose on these points even when the disclosure is negative.

Sample size	We included 54 patient DNA samples at the diagnosis of AML. due to the availability of patient samples, 30 samples were analyzed with 330ng input for superRCA assay and 100nd for ddPCR assay., while 24 patient samples were analyzed with 33ng DNA input both for ddPCR and superRCA assay.
Data exclusions	No data were excluded from the analysis.
Replication	We performed three replicated measurements for the spike-in samples with superRCA assay and two replicates for the ddPCR analysis of the spike-in samples. All the replicates from superRCA and ddPCR measurements were reproduced successfuly. For the superRCA assays, we have been running the assay on different targets on daily bases, we observed a reproducible assay on those established assays. The same patient samples were used to compare the analytical performance between ddPCR and superRCA assay.
Randomization	We prepared the same set of patient DNA samples for the side by side comparasion of superRCA and ddPCR assays. The sample ID were randomized by our clinical collegues before they were hand over to us for the superRCA analysis.
Blinding	Yes, the patient sample were analyzed in blinded fashion.

Reporting for specific materials, systems and methods

We require information from authors about some types of materials, experimental systems and methods used in many studies. Here, indicate whether each material, system or method listed is relevant to your study. If you are not sure if a list item applies to your research, read the appropriate section before selecting a response.

Materials & experimental systems

n/a	Involved in the study	
x		Antibodies
x		Eukaryotic cell lines
x		Palaeontology and archaeology
x		Animals and other organisms
	x	Human research participants
x		Clinical data
x		Dual use research of concern

Methods

n/a	Involved in the study	
x		ChIP-seq
	x	Flow cytometry
x		MRI-based neuroimaging

Human research participants

Policy information about studies involving human research participants

Population characteristics	Uppsala and Stockholm cohort included 54 patients with myeloid neoplasias (Acute Myeloid Leukemia ((AML) including secondary AML and 1 case of Acute Promyelocytic Leukemia (APL)), Myelodysplastic Syndrome (MDS) and myeloprolifeative neoplasia (MPN). The cohort comprised 23 females (43%) and 31 males (57%) and patients had a median age of 70,5 years (range 26-87).
Recruitment	Participants were recruited solely based on prevalence of selected mutations. Mutational data was known from clinical diagnostic workup or prior mutational analysis, all the participants of this study were informed and consent was obtained from every participants. There is no financial compensation made to the participants of this study.
Ethics oversight	This study was approved by the Regional Ethics Committee of Uppsala-Örebro and Stockholm (2014/233, 2019/00130 and 2017/2085-31/2).

Note that full information on the approval of the study protocol must also be provided in the manuscript.

Flow Cytometry

Plots

Confirm that:

- [x] The axis labels state the marker and fluorochrome used (e.g. CD4-FITC).
- [x] The axis scales are clearly visible. Include numbers along axes only for bottom left plot of group (a 'group' is an analysis of identical markers).
- [x] All plots are contour plots with outliers or pseudocolor plots.
- [x] A numerical value for number of cells or percentage (with statistics) is provided.

Methodology

Sample preparation	The superRCA products are nucleic acid products produced with superRCA protocol, it has nothing to do with standard antibody based flow experiment.
Instrument	B&C CytoFlex
Software	CytExpert 2.4.0.8 were used to analyze the data.
Cell population abundance	The superRCA products located either in wild-type or mutant gates.
Gating strategy	Not applicable for superRCA products.

- [x] Tick this box to confirm that a figure exemplifying the gating strategy is provided in the Supplementary Information.
